# Ursodeoxycholic Acid Ameliorates Fructose-Induced Metabolic Syndrome in Rats

**DOI:** 10.1371/journal.pone.0106993

**Published:** 2014-09-09

**Authors:** Amr A. A. Mahmoud, Shimaa M. Elshazly

**Affiliations:** Department of Pharmacology, Faculty of Pharmacy, Zagazig University, Zagazig, Egypt; Bambino Gesu' Children Hospital, Italy

## Abstract

The metabolic syndrome (MS) is characterized by insulin resistance, dyslipidemia and hypertension. It is associated with increased risk of cardiovascular diseases and type-2 diabetes. Consumption of fructose is linked to increased prevalence of MS. Ursodeoxycholic acid (UDCA) is a steroid bile acid with antioxidant, anti-inflammatory activities and has been shown to improve insulin resistance. The current study aims to investigate the effect of UDCA (150 mg/kg) on MS induced in rats by fructose administration (10%) in drinking water for 12 weeks. The effects of UDCA were compared to fenofibrate (100 mg/kg), an agonist of PPAR-α receptors. Treatment with UDCA or fenofibrate started from the 6^th^ week after fructose administration once daily. Fructose administration resulted in significant increase in body weight, elevations of blood glucose, serum insulin, cholesterol, triglycerides, advanced glycation end products (AGEs), uric acid levels, insulin resistance index and blood pressure compared to control rats. Moreover, fructose increased oxidative stress in aortic tissues indicated by significant increases of malondialdehyde (MDA), expression of iNOS and reduction of reduced glutathione (GSH) content. These disturbances were associated with decreased eNOS expression, increased infiltration of leukocytes and loss of aortic vascular elasticity. Treatment with UDCA successfully ameliorated the deleterious effects of fructose. The protective effect of UDCA could be attributed to its ability to decrease uric acid level, improve insulin resistance and diminish oxidative stress in vascular tissues. These results might support possible clinical application of UDCA in MS patients especially those present with liver diseases, taking into account its tolerability and safety. However, further investigations on human subjects are needed before the clinical application of UDCA for this indication.

## Introduction

The metabolic syndrome (MS) is a pathological condition characterized by obesity, insulin resistance, dyslipidemia, atherosclerosis and hypertension [1], [2]. The metabolic disturbance associated with this syndrome has been shown to increase the risk of developing cardiovascular diseases and type-2 diabetes [3], [4]. These deleterious influences raised worldwide attention to the MS and to the possible strategies for its prevention [5].

The increased consumption of fructose, commonly used in processed food and soft drinks, is one of the most important factors contributing to the growing prevalence of the MS [6]. Experimentally, fructose overload in animals can result in insulin resistance, hyperinsulinemia, hypertriglyceridemia, impaired glucose tolerance and can raise blood pressure [7], [8]. These metabolic disturbances bear a resemblance to the human MS [9], [10]. Therefore, the model of fructose-drinking rats is being widely used to induce the MS independent of obesity or genetic contributions and to study the possible interventions [11].

Hyperuricemia and oxidative stress have been proposed as causative factors in the development of insulin resistance, hyperinsulinemia and the progression of MS in rats induced by fructose consumption [10], [12]. In addition, a causal link has been suggested to exist between insulin resistance/hyperinsulinemia and the development of hypertension in this model [10]. This link has been explained by different mechanisms such as increased activity of vasoconstrictors [13], [14], activation of the sympathetic nervous system [15] and impaired endothelium-dependent relaxation [16]. It has been reported that the development hypertension in fructose-drinking rats model can be prevented by inhibiting any one of these factors, with varied effects on insulin sensitivity [10].

Ursodeoxycholic acid (UDCA) is a steroid bile acid that has been widely used for the treatment of chronic cholestatic liver diseases [17]. Different mechanisms have been proposed for the actions of UDCA, including anti-inflammatory, anti-apoptotic, membrane stabilizing, antioxidant and immunomodulating effects [18], [19]. In addition, it has been reported that UDCA possesses a cardiovascular protective effect [20]. Recently, Ratziu and coworkers [21] reported that high-dose of UDCA improved glycemic parameters and insulin resistance in patients with nonalcoholic steatohepatitis. Furthermore, it has been shown that UDCA improved insulin resistance and hepatic steatosis in animals fed a high-fat diet [22]. In addition, Sinisalo and his colleagues showed that treatment of coronary heart disease patients with depressed endothelial function using UDCA could enhance NO-independent endothelial vasodilatation [23]. Taken together, these reports withdrew our attention for the probable effect of UDCA in preventing the MS and the associated vascular endothelial impairment induced by fructose in rats.

Therefore, the current study aims to investigate the effect of UDAC to prevent or ameliorate the MS in a model of fructose-drinking rats. In this attempt, we compared the effects of UDAC with fenofibrate, a peroxisome proliferator activated receptor (PPAR)-α ligand, which is used widely for the treatment of hypertriglyceridemia [24] and has been shown to exert a protective effect in MS patients [25].

## Materials and Methods

### Ethics Statement

Experimental design and animal handling procedures were approved by the local authorities, Ethical Committee for Animal Handling at Zagazig University (ECAHZU), at the Faculty of Pharmacy, Zagazig University, Egypt in accordance with the recommendations of the Weatherall report. Every effort was done to minimize the number of animals and their suffering.

### Animals

Adult male Wistar rats (140–160 g) were obtained from the Faculty of Veterinary Medicine, Zagazig University, Egypt. Rats were acclimatized for one week before starting experiments. They were housed in stainless steel cages (three rats/cage) and kept at controlled temperature (23±2°C), humidity (60±10%) and light/dark (12/12 h) cycle. Rats were supplied with commercially available normal chow diet and water ad libitum.

### Drugs

Fructose was purchased from El-Nasr Chemical Co. (Cairo, Egypt). Fenofibrate was supplied from Abbott (Egypt) and UDAC from Minapharm (Egypt). All other chemicals were of analytical grade. Drugs were suspended in 1% gum acacia in distilled water immediately before administration.

### Experimental design

The animals were randomly divided into four experimental groups (n = 5–8). Group 1 (Control): rats received vehicle only (1% gum acacia); group 2 (FDR): rats received the vehicle and administered fructose (10%) in drinking water for 12 weeks; group 3 (UDCA): rats administered fructose (10%) in drinking water for 12 weeks plus UDCA (150 mg/kg) suspended in the vehicle; group 4 (FENO): rats administered fructose (10%) in drinking water for 12 weeks plus fenofibrate (100 mg/kg) suspended in the vehicle. Treatment with UDCA and fenofibrate started from the 6^th^ week after fructose administration once daily every morning at 10:00 AM by oral gavage.

### Determination of blood glucose

After the last dose of drugs, rats were fasted overnight. Fasting blood glucose was determined with an automatic blood glucose meter (Super Glucocard, Japan) using blood samples from the tail tip.

### Blood pressure measurement

Rats were anaesthetized with urethane (120 mg/100 g, i.p.) given as 25% freshly prepared solution. A cut open was made in the skin of the neck region, and a slit incision was made in the platysma muscles. After the identification of trachea, a small incision was made on the cartilage, and tracheostomy was done using a rodent tracheal intubation tube. The carotid artery was exposed and cannulated using a fine polyethylene catheter filled with heparinised saline (100 IU/ml) and connected to a pressure transducer “PT400”. Systolic blood pressure (SBP) and diastolic blood pressure (DBP) were then recorded using Oscillograph 400 MD 4C-Palmer (Bioscience, Washington, USA). Mean arterial pressure (MAP) was calculated as follow: MAP  =  ⅓ SBP + ⅔ DBP.

### Collection of blood samples and serum separation

After recording of blood pressure, blood samples weretaken out by cardiac puncture and then centrifuged for 20 min. at 3,000 g. Serum was stored at −20°C and thawed just before use for the determination of cholesterol, triglycerides, uric acid, advanced glycation end products (AGEs) and insulin levels.

### Isolation of aortic sections

After blood collection, descending thoracic aorta was carefully excised after cleaning of excess connective tissues and fats. Isolated aorta was then cut into rings (2–3 mm length); some rings were rapidly stored in 10% phosphate-buffered formalin solution at room temperature for histopathological examination. The remaining aortic rings were immersed immediately in liquid nitrogen and kept at −80°C for the determination of GSH and MDA contents, as well as the expression of iNOS, eNOS and xanthine oxidase (XO).

### Biochemical analysis

#### Determination of total cholesterol, triglycerides and uric acid levels

Serum total cholesterol, triglycerides and uric acid were determined using colorimetric kits (Biodignostic, Cairo, Egypt) using a UV-visible spectrophotometer (UV-1601PC, Shimadzu, Japan) following the manufacturer's instructions.

#### Determination of AGEs level

Serum level of AGEs was determined as described by Sampathkumar et al. [26]. In brief, serum aliquot was diluted in phosphate-buffered saline (1∶50) and the fluorescence was determined (λ_excitation_ = 370 nm, λ_emission_ = 440 nm) using LS45 spectrofluorometer (PerkinElmer Inc., USA). The fluorescence intensity is directly proportional to the concentration of the AGEs in serum samples.

#### Determination of insulin level

Serum insulin level was measured by sandwich enzyme-linked immunosorbent assay (ELISA) using kits supplied by Millipore (Cairo, Egypt), following the manufacturer's instructions with microtiter plate coated with mouse monoclonal anti-rat insulin antibodies.

#### Calculation of insulin resistance

Insulin resistance was determined using the homeostasis model assessment index for insulin resistance (HOMA-IR) using the following formula: HOMA-IR index  =  [fasting glucose (mmol/L) × fasting insulin (µU/ml)]/22.5 as described by Matthews et al. [27].

#### Determination of oxidative stress markers in aortic sections

Aortic MDA content was determined colorimetrically as described by Satoh [28], using diagnostic kit supplied by Biodiagnostic, Egypt, following the manufacturer's instructions. The assay is based on the reaction between MDA in the homogenized tissue samples with thiobarbituric acid forming a pink adduct, whose absorbance can be measured at 534 nm. Aortic reduced GSH content was determined colorimetrically as described by Beutler et al. [29], using a diagnostic kit supplied by Biodiagnostic, Egypt, following the manufacturer's instructions. The assay is based on the reduction of 5,5′ dithiobis (2-nitrobenzoic acid) with GSH in the sample to produce a yellow product, whose absorbance can be measured at 405 nm.

#### Determination of iNOS, eNOS and xanthine oxidase gene expression using real time PCR

Total RNA was extracted from aorta using Trizol reagent (Invitrogen, Carlsbad, CA) according to the manufacturer's instruction. The RNA pellet was resuspended in DEPC-treated water. The quality and concentration of the RNA was assessed using the OD 260/280 ratio, and only samples with ratio above 1.5 were used in the experiments. Total RNA was reverse transcribed using RevertAid Premium Reverse Transcriptase-Kit (Fermentas International Inc., Burlington, Canada). Briefly, RevertAid H Minus M-MuLV Reverse Transcriptase was added to dNTP Mix (10 mM), 5× Reaction Buffer and random hexamer primers, the mixture was subjected to cDNA synthesis cycling conditions at 37°C for 30 min. and at 85°C for 5 min. Real-time quantitative polymerase chain reaction (RT-qPCR) was performed using ABI prism 7500 sequence detector system (Applied Biosystems, Foster City, CA), using the Maxima SYBR Green qPCR Kit (Fermentas International Inc., Burlington, Canada). Primer sequences were as follow: for iNOS gene, forward: 5′-CGGTTCACAGTCTTGGTGAAAG-3′, reverse: 5′-CAGGTGTTCCCCAGGTAGGTAG-3′; for eNOS gene, forward: 5′-CATACAGAACCCAGGATGGGCT-3′, reverse: 5′ TCCTCAGGAGGTCTTGCACATA-3′; for XO gene, forward: 5′-TGCCATTGATATTGGACAAGTAG-3′, reverse: 5′-TGCAGCTCCTCCATAGTGAA-3′ and for GAPDH gene, forward: 5′ TGCTGGTGCTGAGTATGTCG 3′, reverse: 5′ TTGAGAGCAATGCCAGCC 3′. Reaction mixtures contained 10 pmol/µL of each primer, 12.5 µl Maxima SYBR mix and 5.5 µl nuclease-free water. An amount of 5 µl of template cDNA was added to each reaction mix. The thermal cycling protocol consisted of 2 min. at 50°C and 10 min. at 95°C, followed by 40 cycles at 95°C for 30 s, 60°C for 30 s and 72°C for 30 s. Data from real-time assays were calculated using the v. 1·7 Sequence Detection Software from PE Biosystems (Foster City, CA). Relative expression of studied genes was calculated using the comparative C_t_ method. All values were normalized to the GAPDH gene expression and reported as fold change over background levels detected in diseased group [30].

#### Histopathological examination

The aorta from each rat was rapidly dissected out and tissue sections were fixed in 10% phosphate-buffered formalin solution at room temperature. After an overnight wash, specimens were dehydrated in graded ethanol, cleared in xylene and embedded in paraffin. Paraffin-embedded tissue sections of aorta (4–5 mm thick) were prepared, mounted on slides and kept at room temperature. Thereafter, slides were dewaxed in xylene, hydrated using graded ethanol, and stained for histopathological examination by hematoxylin and eosin (H&E) stain and Weigert's stain for elastic fibers. The sections were examined under light microscope and photographed with a digital camera (Canon, Japan).

### Statistical analysis

All data were expressed as mean ± standard error of the mean (SEM). Statistical analysis was performed using Graphpad prism software v.5 (GraphPad Software Inc., La Jolla, CA, USA). The statistical significance of differences between groups was tested using one-way analysis of variance (ANOVA) followed by Tukey's Post-test. A significant difference was assumed for values of P<0.05.

## Results

### Effect on body weight, serum parameters and blood pressure

As shown in Table 1, fructose administration resulted in a significant increase in the percentage of body weight gain compared to control rats (27.8%±0.6 vs. 20.9%±1.0 increase from basal weight, P<0.001). After 12 weeks, FDR showed significant increases in fasting blood glucose (P<0.001), serum insulin (P<0.001), total cholesterol (P<0.001), triglycerides (P<0.001), AGEs (P<0.001) and uric acid (P<0.001) levels compared to the control group. In addition, significant increases in HOMA-IR (P<0.001), SBP (P<0.001), DBP (P<0.01) and MAP (P<0.001) were observed in FDR compared to the control rats. Treatment of FDR with either UDCA or fenofibrat significantly decreased the percentage increase in body weight compared to rats administered fructose alone (P<0.05, P<0.001, respectively). In addition, administration of UDCA or fenofibrat significantly reduced the elevated fasting blood glucose (P<0.001), serum insulin (P<0.001 and P<0.01, respectively), total cholesterol (P<0.05, P<0.001, respectively), triglycerides (P<0.001), AGEs (P<0.01) and uric acid levels (P<0.001) compared to rats administered fructose alone. Moreover, HOMA-IR index, SBP, DBP and MAP were significantly reduced (P<0.001) by treatment with UDCA or fenofibrat compared to rats administered fructose alone (Table 1).

**Table 1:** Effect of ursodeoxycholic acid (150 mg/kg, p.o.) and fenofibrate (100 mg/kg, p.o.) on body weight, serum parameters and blood pressure in rats administered fructose.

Parameters	Groups			
	Control	FDR	UDCA	FENO
**1. Δ Body weight (%)^a^**	20.9±1.0	27.8±0.6*	24.3±0.8#	22.3±0.8#
**FBG (mmol/l)**	5.1±0.09	7.6±0.41*	5.8±0.22#	5.7±0.17#
**Insulin (µU/ml)**	9.2±0.66	20.1±1.9*	10.6±0.65#	12.7±0.6#
**HOMA-IR**	2.1±0.19	6.9±0.99*	2.7±0.14#	3.2±0.19*#
**T. CH (mg/dl)**	43.5±3.9	78.6±5.6*	56.6±4.5#	43.5±4.8#
**TG (mg/dl)**	57.7±4.3	117.9±3.9*	55.5±4.3#	52.12±4.7#
**AGEs (pg/mg prot.)**	48.9±2.6	77.1±5.9*	52.4±4.2#	52.7±4.9#
**Uric acid (mg/dl)**	0.37±0.07	2.42±0.1*	1.1±0.12#	0.82±0.09#
**SBP (mmHg)**	105±2.4	140±5.9*	103±4.2#	86±4.3#
**DBP (mmHg)**	72.3±1.6	100±5.7*	70±3.3#	53.7±5.7#
**MAP (mmHg)**	86.5±1.3	113.3±5.6*	81.0±3.4#	64.6±5.1#

Values are expressed as mean ± SEM (n = 5–8). **^a^**Percentage change in body weight from basal value before starting the experiment. ^*^P<0.05 vs. control, ^#^P<0.05 vs. FDR. FDR, fructose-drinking rats; UDCA, fructose-drinking rats treated with ursodeoxycholic acid (150 mg/kg, p.o.); FENO, fructose-drinking rats treated with fenofibrate (100 mg/kg, p.o.). FBG, fasting blood glucose; HOMA-IR, homeostasis model assessment index for insulin resistance; T. CH, total cholesterol; TG, triglycerides; AGEs, advanced glycation end products; prot., protein; SBP, systolic blood pressure; DBP, diastolic blood pressure; MAP, mean arterial pressure.

### Oxidative stress markers

**Figure 1:**
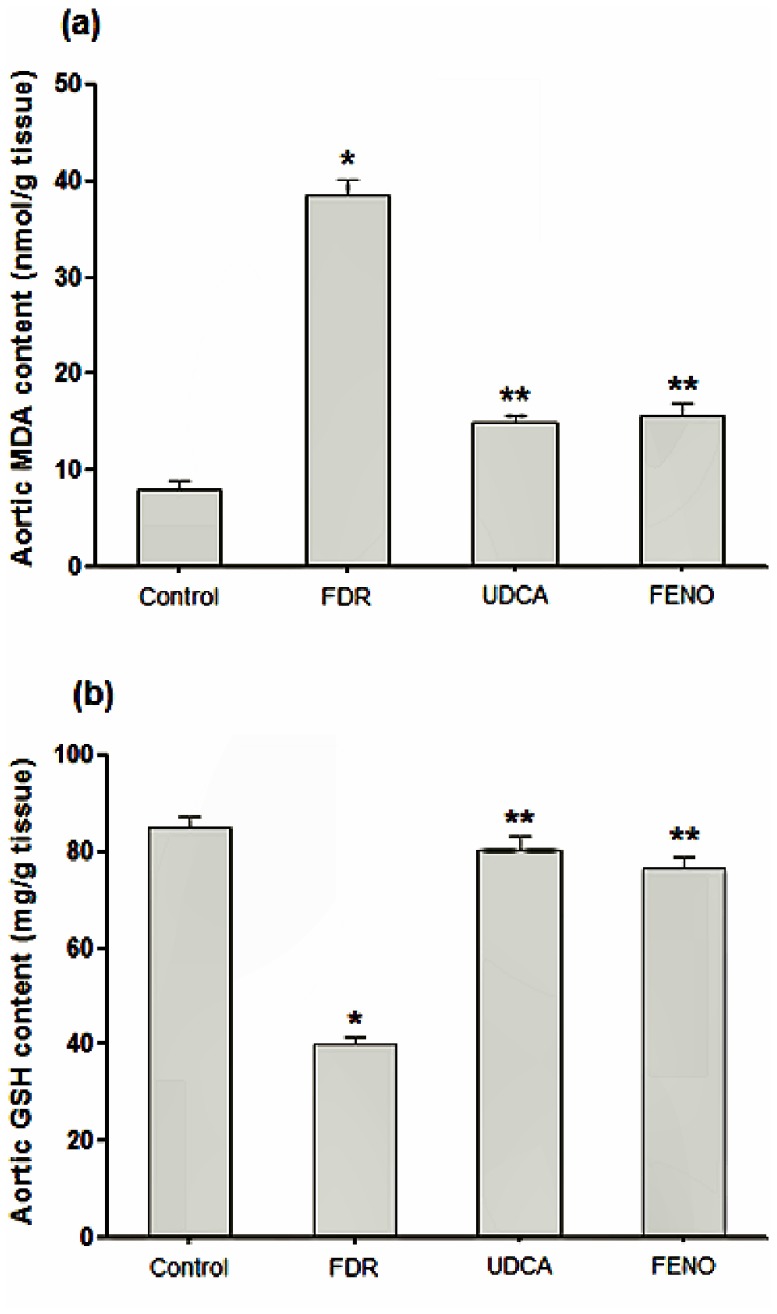
Effect of ursodeoxycholic acid (150 mg/kg, p.o.) and fenofibrate (100 mg/kg, p.o.) on aortic malondialdehyde (MDA) content (a) and aortic reduced glutathione (GSH) content (b) in fructose-drinking rats. (n = 5–8). ^*^P<0.05 vs. control, ^**^P<0.05 vs. FDR. FDR, fructose-drinking rats; UDCA, fructose-drinking rats treated with ursodeoxycholic acid (150 mg/kg, p.o.); FENO, fructose-drinking rats treated with fenofibrate (100 mg/kg, p.o.).

Aorta isolated from FDR showed a significantly elevated MDA content compared to control rats (38.46±1.68 vs. 8.05±0.78 nmol/g. tissue, P<0.001). This elevation was significantly diminished by treatment with either UDCA (14.95±0.74 vs. 38.46±1.68 nmol/g. tissue, P<0.001) or fenofibrate (15.58±1.39 vs. 38.46±1.68 nmol/g. tissue, P<0.001) as depicted in Fig. 1a. Another important marker of oxidative stress, reduced GSH, was significantly reduced in aortic sections isolated from rats administered fructose for 12 weeks compared to the control group (40.09±1.51 vs. 84.99±2.28 mg/g. tissue, P<0.001). Administration of UDCA or fenofibrate to FDR significantly increased the level of GSH (80.46±2.94 and 76.66±2.29 vs. 40.09±1.51 nmol/g. tissue, respectively, P<0.001) and restored it close to the control levels (P>0.05) as shown in Fig. 1b.

### Effect on aortic expression of iNOS, eNOS and XO

**Figure 2:**
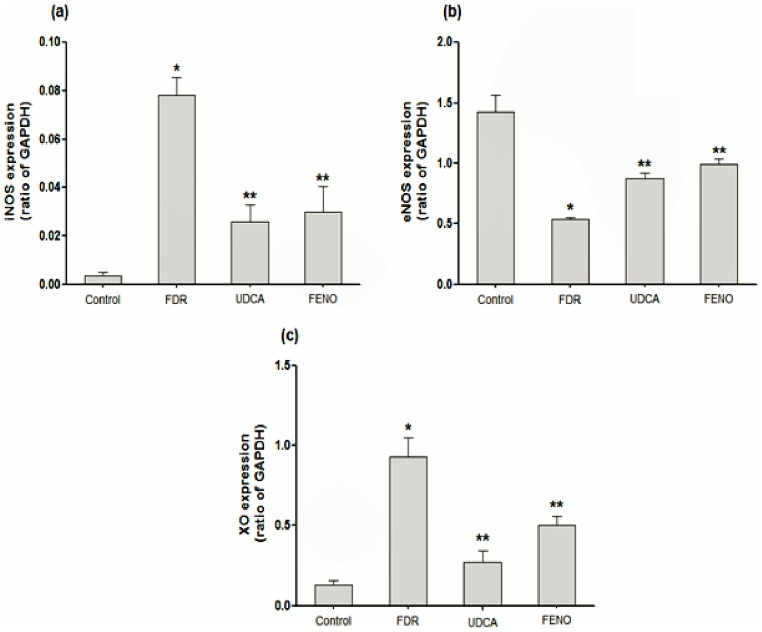
Effect of ursodeoxycholic acid (150 mg/kg, p.o.) and fenofibrate (100 mg/kg, p.o.) on gene expression of iNOS (a); eNOS (b) and XO (c) of aortic sections isolated from fructose-drinking rats. (n = 5–8). ^*^P<0.05 vs. control, ^**^P<0.05 vs. FDR. FDR, fructose-drinking rats; UDCA, fructose-drinking rats treated with ursodeoxycholic acid; FENO, fructose-drinking rats treated with fenofibrate. iNOS, inducible nitric oxide synthase; eNOS, endothelial nitric oxide synthase; XO, xanthine oxidase; GAPDH, glyceraldehyde 3-phosphate dehydrogenase.

As depicted in Fig. 2, fructose consumption for 12 weeks induced significant reductions in the expression of eNOS (P<0.001), while it induced significant increases in the expression of iNOS (P<0.001) and XO (P<0.001) compared to the control group. Alterations of gene expression induced by fructose were ameliorated by treatment with UDCA or fenofibrate. Both drugs showed comparable effects resulting in a significant elevation of eNOS expression (P<0.05, P<0.01, respectively), significant reductions of iNOS expression (P<0.001, P<0.01, respectively) and XO expression (P<0.001, P<0.01, respectively) compared to FDR.

### Histopathological examination

**Figure 3:**
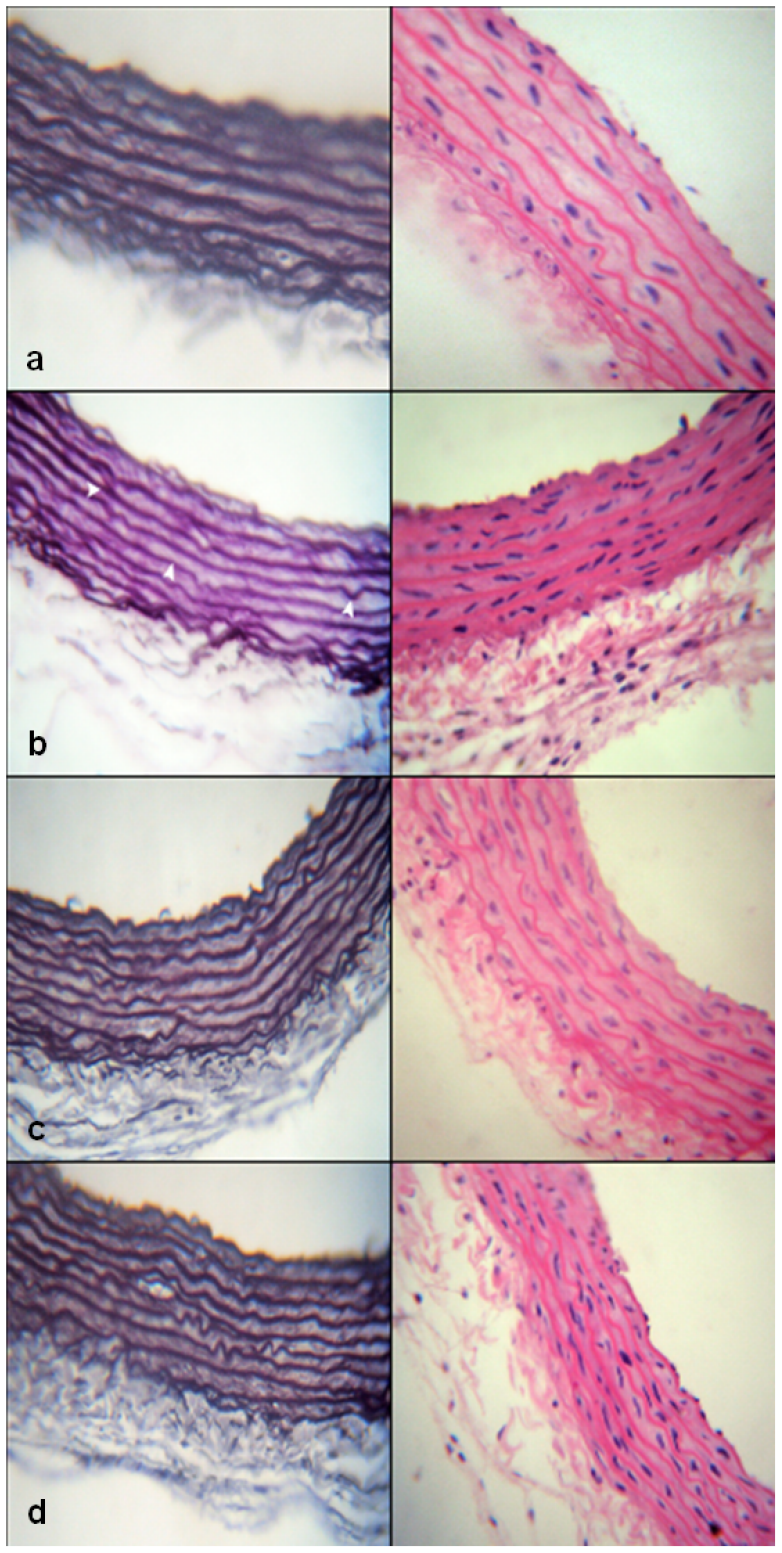
Histological examination of aortic sections stained with H&E (×400) on the right panel and Weigert's elastic stain (×400) on the left panel. Representative aortic sections were obtained from control rats (a); FDR (b); FDR administered UDCA 150 mg/kg (c) and FDR administered fenofibrate 100 mg/kg (d). White arrowheads point to the relatively straight elastic laminae of aorta from FDR. FDR, fructose-drinking rats; UDCA, ursodeoxycholic acid.

As depicted in Fig. 3a, staining aortic sections of control rats with H&E and Weigert's stain (elastic stain), respectively, revealed normal tunica adventitia with no apparent infiltration of leukocytes and wavy elastic fibers in tunica media. On the other hand, aortic sections from FDR showed multiple leukocytes infiltrating the tunica adventitia and elastic fibers that are relatively straighter (Fig. 3b) compared to the control rats. In rats administered UDCA (Fig. 3c) or fenofibrate (Fig. 3d), no infiltrating leukocytes were detected, while few straight and mostly wavy elastic fibers were observed.

## Discussion

Metabolic syndrome (MS) comprises a constellation of disturbances such as abdominal obesity, insulin resistance and dyslipidemia, which eventually result in hypertension. These disturbances put the patient at a high risk of developing type-2 diabetes and cardiovascular morbidities [3], [31]. The consumption of fructose, among others dietary changes, has been linked to the escalated incidence of MS [32], [33]. This represents a major health risk because fructose intake has greatly increased over the last decades [34]. Experimentally, Fructose has been widely accepted as an animal model for MS that mimics the symptoms afflicting human subjects [9], [10].

Ursodeoxycholic acid (UDCA) is a hydrophilic bile acid used in the treatment of liver diseases such as primary biliary cirrhosis and cystic fibrosis-related cholestasis [35], [36]. Some reports described that UDCA can improve insulin resistance in patients with nonalcoholic steatohepatitis [21]. In addition, another report demonstrated that the taurine conjugate form of UDCA, tauroursodeoxycholic acid, could enhance insulin sensitivity in obese, insulin-resistant subjects [37]. Combined with its ability to reduce triglycerides, these data suggest a possible potential role of UDCA in ameliorating symptoms of the MS. Therefore, in the present study we aim to evaluate the effect of UDCA on fructose-induced MS and associated vascular endothelial impairment in rats. In this regard, the effects of UDCA were compared to fenofibrate. Our selection for fenofibrate as a reference drug is based on previous reports showing that PPAR-α agonists lowered triglycerides level and improved insulin sensitivity in different animal models including rats fed high-fat diet [38], rats fed high-fructose diet [39] and Zucker obese rats [40]. In addition, fenofibrate has been reported to inhibit vascular inflammation in experimental animals [41] and to improve endothelial function and arterial stiffness in obese glucose tolerant men [42].

Our results showed that fructose administration (10%) to rats in drinking water for 12 weeks induced the classic symptoms of the MS. Rats showed significant increase in weight gain compared to the control group. They also had significantly higher levels of blood glucose, serum insulin, total cholesterol, triglycerides, higher SBP, DBP and MAP compared the control rats that did not receive fructose. In addition, fructose induced insulin resistance as evidenced by the significant increase in HOMA-IR index. Together, these alterations confirm the proper induction of MS in our study, which is in agreement with previous reports [8], [43], [44].

Our results showed that fructose administration resulted in hyperuricemia, which is consistent with other previous reports [12], [45], [46]. Fructose-induced hyperuricemia might be attributed to the stimulation of AMP deaminase activity triggered by fructose metabolism resulting in increased formation of uric acid [47]. High level of uric acid during fructose consumption has been suggested as an important causative factor in the development of MS. This is supported by previous findings demonstrating that reducing uric acid level, using XO inhibitors or uricosuric agents, could prevent the development of MS signs in fructose-fed rats [46], [48].

Hyperuricemia plays a pivotal role in the development of insulin resistance and hypertension during MS through uric acid-induced endothelial dysfunction. Nakagawa and coworkers [48] showed that uric acid can block acetylcholine-mediated arterial dilation in a dose-dependent manner suggesting that uric acid can impair endothelial function. Furthermore, it has been shown that uric acid can reduce endothelial NO bioavailability in both cell culture and in experimental animal models [49]. In this context, our results show that fructose-induced hyperuricemia is associated with a significant reduction in the expression of eNOS in thoracic aorta sections. This reduction can contribute to insulin resistance through the inhibition of insulin-stimulated glucose uptake in skeletal muscle, which is actually mediated by increasing blood flow through a NO-dependent pathway [50]. Moreover, reduced production of endothelial NO cause the vascular smooth muscle cells to become more sensitive to the effects of vasoconstrictors, which can result in increased vascular tone and elevated blood pressure [10]. In this regard, our histopathological findings showed that the aortic sections from rats administered fructose lost some of its elasticity as shown by the relative straightening of elastic fibers in tunica media (Fig. 3b) compared to the control rats (Fig. 3a).

Another important causative factor that might be involved in the progression of fructose-induced impairment of vascular endothelium associated with insulin resistance is oxidative stress. The results of the present study demonstrate that fructose consumption increased oxidative stress in aortic tissues as evidenced by the increased production of MDA, GSH and increased expression of iNOS (Fig. 1a, 1b and 2a, respectively). Our results are in agreement with previous reports [51], [52]. Although increased expression of iNOS can result in increased production of NO in aortic tissues, however, under conditions of oxidative stress, superoxide radical is produced which then can interact with NO. This interaction can result in the degradation of NO and the formation of peroxynitrite, a highly toxic oxidant to the vascular tissues [53], [54]. Two main sources of ROS in vascular tissues have been described, namely NADPH oxidase and XO [55], [56]. Indeed, our results showed that the expression of XO in aortic rings from FDR was significantly higher compared to the control rats serving as a continued source of ROS production. In addition, we detected significant leukocyte infiltration in the tunica adventitia of aortic rings from FDR during the histopathological examination. These inflammatory cells play an important role in the consequent vascular dysfunction through increasing the production of ROS primarily by stimulating NADPH oxidase within the vasculature [57]. Our results are consistent with other reports showing also vascular inflammation induced by fructose [58], [59]. We found also that the vascular inflammation and endothelial impairment induced by fructose were not only confined to the aortic vasculature, but also were apparent in kidneys. Renal interstitium inflammation was manifested by the presence of lymphocytes and monocytes, as well as enhanced deposition of collagen fibers around venules in the renal cortex (data not published).

Moreover, we found that FDR had significantly higher levels of serum AGEs compared to the control rats (Table 1). Our results are in harmony with previous results showing that AGEs increase not only under hyperglycemic states, but also by fructose consumption [60], [61]. Fructose-induced elevation of AGEs level has many deleterious consequences on the vasculature including cross-linking of collagen, resulting in loss of elasticity and endothelial impairment. In addition, AGEs have been shown to quench NO in vitro and induce inflammation as well as oxidative stress that eventually can lead to vascular impairment and hypertension [62], [63].

Treatment of FDR with UDCA significantly diminished the percentage of body weight gain and ameliorated fructose-induced hyperglycemia, hyperinsulinemia, hypertriglyceridemia, hypercholesterolemia, hypertension, and improved insulin resistance as well (Table 1). The effects of UDCA were comparable to fenofibrate with relatively better effects regarding serum insulin and insulin resistance index. However, this difference does not reach statistical significance.

The protection exerted by UDCA might be attributed to several mechanisms including reduction of uric acid, antioxidant, anti-inflammatory effects and reduction of AGEs. On one hand, our results demonstrated that UDCA significantly reduced serum uric acid level compared to FDR. This reduction was associated with a significant improvement in the aortic expression of eNOS and aortic wall elasticity denoted by the presence of many wavy elastic fibers (Fig. 3c). UDCA-induced reduction of uric acid might be due reduced expression of XO, as we could find such an effect in aortic sections from rats treated with UDCA. In support to this postulation, Sokolovic et al., demonstrated also that UDCA can reduce XO activity in liver tissues [64]. On the other hand, UDCA exhibited remarkable antioxidant effect showed by significant reduction in aortic MDA content and expression of iNOS as well as significant increase in reduced GSH content (Fig. 1a, 1b and 2a, respectively). Moreover, UDCA reduced vascular endothelial inflammation as demonstrated by the absence of infiltrating leukocytes in the aortic tissues (Fig. 3c). UDCA also reduced the formation of AGEs, through improving insulin resistance, which may be involved also in reducing oxidative stress and vascular endothelial inflammation. All these effects help in ameliorating signs of MS and normalizing blood pressure.

These findings seem interesting talking into consideration the added therapeutic benefit of using UDCA in patients with non-alcoholic fatty liver disease (NAFLD), which is considered as a hepatic component of MS and diabetes mellitus type-2 and the most common cause of hepatic dysfunction [65]. Although the protective effects of UDCA observed in this study were almost comparable to fenofibrate, however, UDCA might be preferable considering safety. Fibrates, although generally well tolerated, may cause myopathy and rhabdomyolysis both in humans [66] and rats [67]. In addition, some case studies described hepatotoxicity associated with fenofibrate [68], [69]. On the other hand, UDCA has been shown to be well tolerated [70] and additionally proven to be safe even when administered during pregnancy [71].

In conclusion, the present study provides an evidence of the potential protective effect of UDCA against MS and associated hypertension induced by fructose in rats. These effects could be attributed to the ability of UDCA to decrease uric acid level and oxidative stress particularly in vascular tissues. In addition, these results may support future possible clinical application of UDCA in MS patients especially those present with NAFLD. However, this assumption needs further investigations on human subjects to be confirmed and clinically applied.
